# Genomic Surveillance of Methicillin-resistant *Staphylococcus aureus*: A Mathematical Early Modeling Study of Cost-effectiveness

**DOI:** 10.1093/cid/ciz480

**Published:** 2019-06-18

**Authors:** Amy Dymond, Heather Davies, Stuart Mealing, Vicki Pollit, Francesc Coll, Nicholas M Brown, Sharon J Peacock

**Affiliations:** 1 York Health Economics Consortium, United Kingdom; 2 London School of Hygiene & Tropical Medicine, United Kingdom; 3 Cambridge University Hospitals National Health Service Foundation Trust, United Kingdom; 4 Department of Medicine, University of Cambridge, United Kingdom

**Keywords:** MRSA, cost-effectiveness, whole-genome sequencing

## Abstract

**Background:**

Genomic surveillance of methicillin-resistant *Staphylococcus aureus* (MRSA) identifies unsuspected transmission events and outbreaks. Used proactively, this could direct early and highly targeted infection control interventions to prevent ongoing spread. Here, we evaluated the cost-effectiveness of this intervention in a model that compared whole-genome sequencing plus current practice versus current practice alone.

**Methods:**

A UK cost-effectiveness study was conducted using an early model built from the perspective of the National Health Service and personal social services. The effectiveness of sequencing was based on the relative reduction in total MRSA acquisitions in a cohort of hospitalized patients in the year following their index admissions. A sensitivity analysis was used to illustrate and assess the level of confidence associated with the conclusions of our economic evaluation.

**Results:**

A cohort of 65 000 patients were run through the model. Assuming that sequencing would result in a 90% reduction in MRSA acquisition, 290 new MRSA cases were avoided. This gave an absolute reduction of 28.8% and avoidance of 2 MRSA-related deaths. Base case results indicated that the use of routine, proactive MRSA sequencing would be associated with estimated cost savings of over £728 290 per annual hospitalized cohort. The impact in total quality-adjusted life years (QALYs) was relatively modest, with sequencing leading to an additional 14.28 QALYs gained. Results were most sensitive to changes in the probability of a MRSA-negative patient acquiring MRSA during their hospital admission.

**Conclusions:**

We showed that proactive genomic surveillance of MRSA is likely to be cost-effective. Further evaluation is required in the context of a prospective study.

## Teaser

The potential value of genomic surveillance of nosocomial pathogens for outbreak detection and investigation is high but will result in an up-front cost. This early model indicates that proactive genomic surveillance of methicillin-resistant *Staphylococcus aureus* is likely to be cost-effective.

Hospital outbreak detection is founded on principles used by John Snow during his investigation of a cholera epidemic [1]. Routine surveillance identifies patients who carry or are infected with specific pathogens, which are reviewed in relation to patient movement data and other information that could link cases. The identification of 2 or more patients positive for the same pathogenic species who are also linked in time and place triggers an assessment of the probability of an outbreak. In the case of bacterial pathogens, such as methicillin-resistant *Staphylococcus aureus* (MRSA), an assessment may incorporate routine data generated by the microbiology laboratory on antibiotic resistance. The pattern of resistance to the range of antibiotics tested in the laboratory is used as a surrogate for bacterial relatedness, although this lacks sensitivity and specificity. If the initial investigation concludes that an outbreak is possible or likely, formal typing methods may be used to define bacterial relatedness. Available typing methods lack sensitivity because of their low power to discriminate between isolates that reside in the same clone, and can erroneously link isolates that are not recently related [2–4]. Ultimately, the confirmation of an outbreak is based on a combination of imperfect evidence and the intuition of experienced infection control staff.

Whole-genome sequencing represents a major advance in the control of multidrug-resistant organisms (or other organisms) that spread between patients, units, and facilities. When combined with patient movement data, this provides a more accurate determination of transmission events and outbreak statuses than standard infection control methods alone [2–7]. An option for the uptake of bacterial sequencing into clinical practice is to simply swap the typing methods used during an outbreak investigation, but there would be several advantages of a process redesign in which sequencing was used proactively [8]. This would begin with the routine sequencing of specific bacterial species of interest and the quantitation of genetic relatedness between isolates of the same species. Following sequencing, the decision to investigate (or not) would be based on a desk-based analysis of the degree of relatedness, combined with information on patient movements. This model would support efficient, highly targeted infection control interventions, provided that data can be generated rapidly and in a format that does not require specialist informatics knowledge. Early evidence for the potential benefits of this “Sequence First” approach came from a study of genomic surveillance of MRSA that was isolated in a large clinical microbiology laboratory in the east of England over 12 months [6]. This led to the identification of hundreds of transmission clusters that were not detected by standard infection controls. This highlights a missed opportunity for greater control through early outbreak detection, followed by action to minimize ongoing transmissions.

The potential value of genomic surveillance of nosocomial pathogens is compelling, but will result in an up-front cost. A rationale to support this cost is to align outbreak detection with other types of activity that relate to safety culture, which are fully justified on the basis that they save lives through prevention, rapid detection, and effective action. However, evidence of cost-effectiveness is often demanded of changes in healthcare practice.

## METHODS

### Model Overview

**Figure 1.**
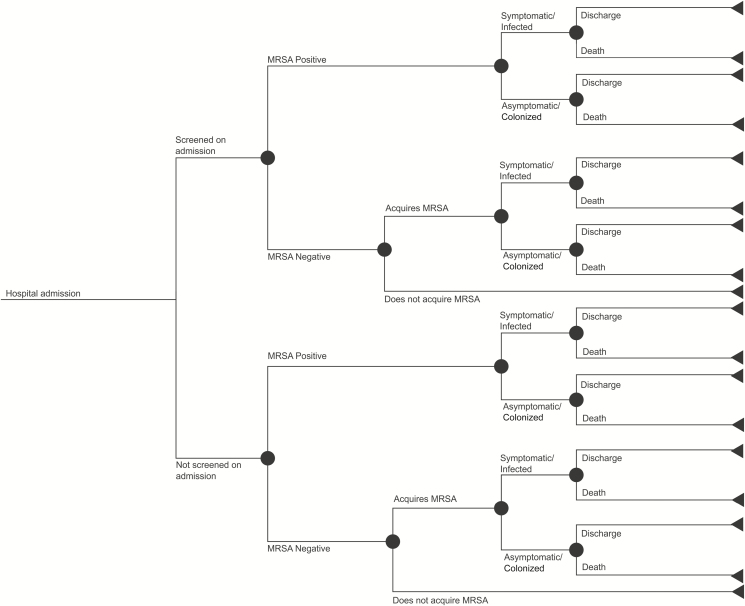
Model schematic. Abbreviation: MRSA, methicillin-resistant *Staphylococcus aureus*.

The aim of the model was to estimate the cost-effectiveness of whole-genome sequencing of MRSA combined with standard infection control practice, versus standard practice alone. A decision tree framework (Figure 1) was used to estimate the reduction in the total number of MRSA acquisitions (including any subsequent infection) and, hence, the cost-effectiveness of whole-genome sequencing over 1 year, based on an annual cohort of newly admitted, hospitalized patients. Patients admitted to hospital or in high-risk specialties may be screened proactively for carriage of MRSA, with additional MRSA testing occurring when clinical samples are taken to investigate patients with clinical features of infection (or as part of an outbreak investigation). Patients within the model were classified as either MRSA-negative (as confirmed by a negative MRSA sample in some cases); MRSA-positive, asymptomatic (screening swab from a carrier); or MRSA-positive, infected (isolated from a clinical sample).

The model was built from the National Health Service (NHS) and personal social services perspective, which is consistent with the National Institute for Health and Care Excellence reference case [9]. As the model only estimated the cost-effectiveness of whole-genome sequencing within 1 annual cohort of hospitalized patients, the discounting of costs and benefits was not necessary. Per-patient quality-adjusted life years (QALYs) were generated, and the primary outcome of the model was the incremental cost per QALY gained.

### Data Sources and Model Parameters

#### Classification of Patients

Data generated during a 12-month prospective observational cohort study between April 2012 and April 2013 were used to inform patient classifications [6]. In brief, the Clinical Microbiology and Public Health Laboratory at the Cambridge University Hospitals NHS Foundation Trust processed samples from consecutive, MRSA-positive individuals. This facility received samples from 3 hospitals and 75 general practices in the east of England. A total of 1465 MRSA-positive patients were identified, and 2282 MRSA isolates underwent whole-genome sequencing. For all MRSA-positive cases, epidemiological data (including hospital ward stays and residential post codes) were recorded. The catchment area from which the study patients were drawn contained a total of 5 012 137 residents, according to the 2011 UK census [6].

#### Effectiveness Inputs

The effectiveness of whole-genome sequencing was based on the relative reduction in the total number of MRSA acquisitions that occurred in a cohort of hospitalized patients in the year following their index admissions. The number of annual MRSA acquisitions expected within current practice (with data from the 12-month observational cohort study used as a proxy) was multiplied by the relative reduction to estimate the reduction in the number of MRSA acquisitions following whole-genome sequencing implementation. Due to a lack of published data to date, the likely size of this relative reduction of MRSA acquisition is hypothetical (Table 1). The probability of a MRSA-negative patient acquiring MRSA whilst in hospital was assumed to be 0.5%, in lieu of forthcoming data from an ongoing clinical evaluation at Cambridge University Hospitals. To explore the implications of these assumptions on the cost-effectiveness of whole-genome screening, both inputs were varied in the sensitivity analyses.

**Table 1.** Model Inputs

Patient Classification Parameters (%)		
Percentage of patients that undergo MRSA screening on admission	87.5	Cambridge University Data
Proportion of patients MRSA positive following screening	1.1	Cambridge University Data
Proportion of patients MRSA positive not screened	1.1	Assumed equal to above due to an absence of data
Probability MRSA-positive patient has symptomatic MRSA versus asymptomatic MRSA	14.1	Cambridge University Data
Effectiveness inputs (%)		
Probability of MRSA-negative patient acquiring MRSA during their stay (CP)	0.5	Assumption
Reduction in MRSA acquisition due to genome sequencing	90	Assumption
Quality of life and mortality inputs		
Quality of life decrement associated with symptomatic MRSA	0.35	US decision analytic model [19]
Proportion of symptomatic MRSA patients that die before discharge due to MRSA infection (%)	4.6	US cost study [10]
Proportion of asymptomatic MRSA patients that die before discharge due to MRSA colonization (%)	0	Assumption
Costs inputs (£)		
Cost per case of symptomatic MRSA	17 238	Kunori et al [12]
Cost per case of asymptomatic MRSA	386.80	Canadian decision tree [11]
Cost per genome sequenced	100	A descriptive study of WGS for MRSA [4]
Cost per screen positive	8.19	UK model. Robotham et al [14]
Cost per screen negative	4.79	UK model. Robotham et al[14]
Cost per clinical sample	3.62	UK model. Robotham et al [14]

Abbreviations: CP, current practice; MRSA, methicillin-resistant *Staphylococcus aureus*; WGS, whole-genome sequencing.

#### *Methicillin-resistant* Staphylococcus aureus*–related Mortality*

Within the early model, we assumed that 4.6% of all symptomatic, infected MRSA inpatients (involving any site or organ) would die of MRSA-related causes before their hospital discharge (Table 1). This value came from a retrospective analysis of US National Inpatient Sample data from 2010 to 2014, where 358 140 MRSA-related hospitalizations were recorded [10]. Consistent with the previous literature [11], we assumed that colonized patients with no infection faced no MRSA-associated mortality risk.

#### Costs

We identified only 1 economic evaluation from a targeted literature search of MRSA surveillance and screening strategies in the United Kingdom, which reported the cost of a MRSA-infected patient when admitted to an intensive care unit [12]. However, they did not report the cost of colonization, and we used a Canadian paper as the source for this parameter [11]. The paper reported the cost per case of positive, asymptomatic MRSA colonization in Canadian dollars, which was converted into British pounds using the exchange rate in 2010. A unit cost of £100 per genome sequenced was used in the base case analysis [4]. The costs to screen a patient for MRSA on admission to hospital, and for a clinical sample to be taken to investigate patients with clinical features of infection (as part of an outbreak investigation), were both taken from the NHS Scotland MRSA Screening Pathfinder Programme [13] (reported in Robotham et al [14]). All unit costs used within the economic model were inflated to the 2017/18 price year using the most recent Pay and Prices Index within the Personal Social Services Research Unit [15] and are presented in Table 1.

#### Health-related Quality of Life 

We applied a health-related quality-of-life decrement of 0.35 to patients within the symptomatic health state to generate lost QALYs (Table 1). As MRSA carriage is asymptomatic, it was assumed that there is no QALY decrement associated with MRSA-positive colonization without infection.

### Sensitivity Analysis

A sensitivity analysis was used to illustrate and assess the level of confidence associated with the conclusions of our economic evaluation. Both 1-way (where input parameters are varied 1 by 1 between plausible extremes) and 2-way (where more than 1 parameter is varied at the same time) sensitivity analyses were conducted to assess the robustness of the overall results within the model and to quantify effects of uncertainty on the cost-effectiveness of whole-genome sequencing.

## RESULTS

### Clinical Outcomes

The clinical outcomes estimated within the early model are presented in Table 2. A cohort of 65 000 patients were run through the model (equating to the estimated number of annual admissions for Addenbrooke’s hospital between April 2012 and April 2013) [6]. Under the assumption that data from whole-genome sequencing would result in a 90% reduction in MRSA acquisition, 290 new MRSA cases were avoided (which includes asymptomatic carriage and clinical infection). This gave an absolute reduction of 28.8% and an avoidance of 2 MRSA-related deaths.

**Table 2.** Clinical Outcomes per Annual Hospitalized Patient Cohort (*N* = 65 000)

Clinical Outcome	WGS + CP	CP	Incremental	
			Absolute	Relative (%)
Number of patients with MRSA sequenced	715	0	715	1.10
Number of MRSA-negative patients that acquire a MRSA infection^a^	5	45	−41	−90
Number of asymptomatic MRSA cases	614	863	−249	−28.8
Number of symptomatic MRSA cases	101	142	−41	−28.8
Number of MRSA-related deaths	5	7	−2	−28.8

As these clinical outcomes are dependent upon assumptions within the model, we would not expect them to be equal to the to the results presented in the aforementioned prospective, observational cohort study [6].

Abbreviations: CP, current practice; MRSA, methicillin-resistant *Staphylococcus aureus*; WGS, whole-genome sequencing.

^a^This refers to patients who were MRSA negative upon admission to hospital.

### Economic Outcomes

**Figure 2.**
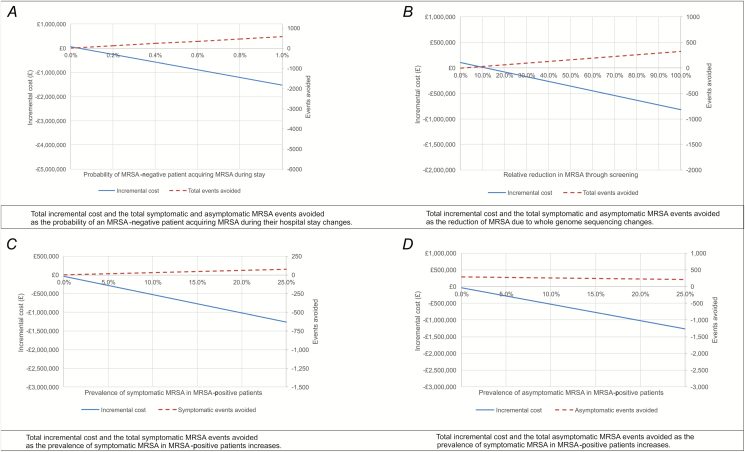
Threshold graphs (cohort of 65 000 patients). A negative incremental cost illustrates whole-genome sequencing as cost saving. For example, a −£1 000 000 incremental cost means whole-genome sequencing is £1 000 000 cheaper than current practice alone. Abbreviation: MRSA, methicillin-resistant *Staphylococcus aureus*.

Our base case results indicated that the use of routine, proactive MRSA sequencing would be associated with estimated cost savings of over £728 290 per annual, hospitalized cohort (Table 3). The difference in total QALYs between the 2 patient cohorts was more modest, with sequencing leading to an additional 14.28 QALYs gained. The results were most sensitive to changes in the probability of a MRSA-negative patient acquiring MRSA during their hospital admission. The results were also sensitive to the proportion of MRSA-positive patients with a clinical infection at the time of presentation to hospital, and the relative reduction in cases of MRSA acquisition due to actions taken in response to whole-genome sequencing data. Figure 2 presents threshold graphs illustrating the changes in incremental costs and MRSA carriage, as well as the clinical infection events avoided as these parameters are changed. The higher the probability of a MRSA-negative patient acquiring MRSA during their stay, the greater the MRSA events avoided and the more costs saved by MRSA sequencing. A greater cost saving was observed as the relative reduction in MRSA acquisitions due to the implementation of sequencing increased. See the Supplementary Materials for a further sensitivity analysis.

**Table 3.** Economic Outcomes per Annual Hospitalized Cohort (*N* = 65 000)

Economic Outcomes	WGS + CP (£)	CP (£)	Incremental	
			Absolute (£)	Percentage (%)
Costs				
Genome sequencing costs	71 466	0.00	71 466	100
MRSA-related treatment costs	1 974 473	2 774 112	−799 639	−40
Admission screening costs	274 462	274 462	0.00	0
Outbreak investigation screening costs	39 108	39 083	24	0
Clinical sampling costs	365	513	−148	−40
Total cost	2 359 873	3 088 170	−728 297	−31
QALYs				
Total QALYS	64 965	64 950	14	0.02
Incremental cost per QALY	Less costly and more effective (dominant)			

Abbreviations: CP, current practice; MRSA, methicillin-resistant *Staphylococcus aureus*; QALY, quality-adjusted life year; WGS, whole-genome sequencing.

**Figure 3.**
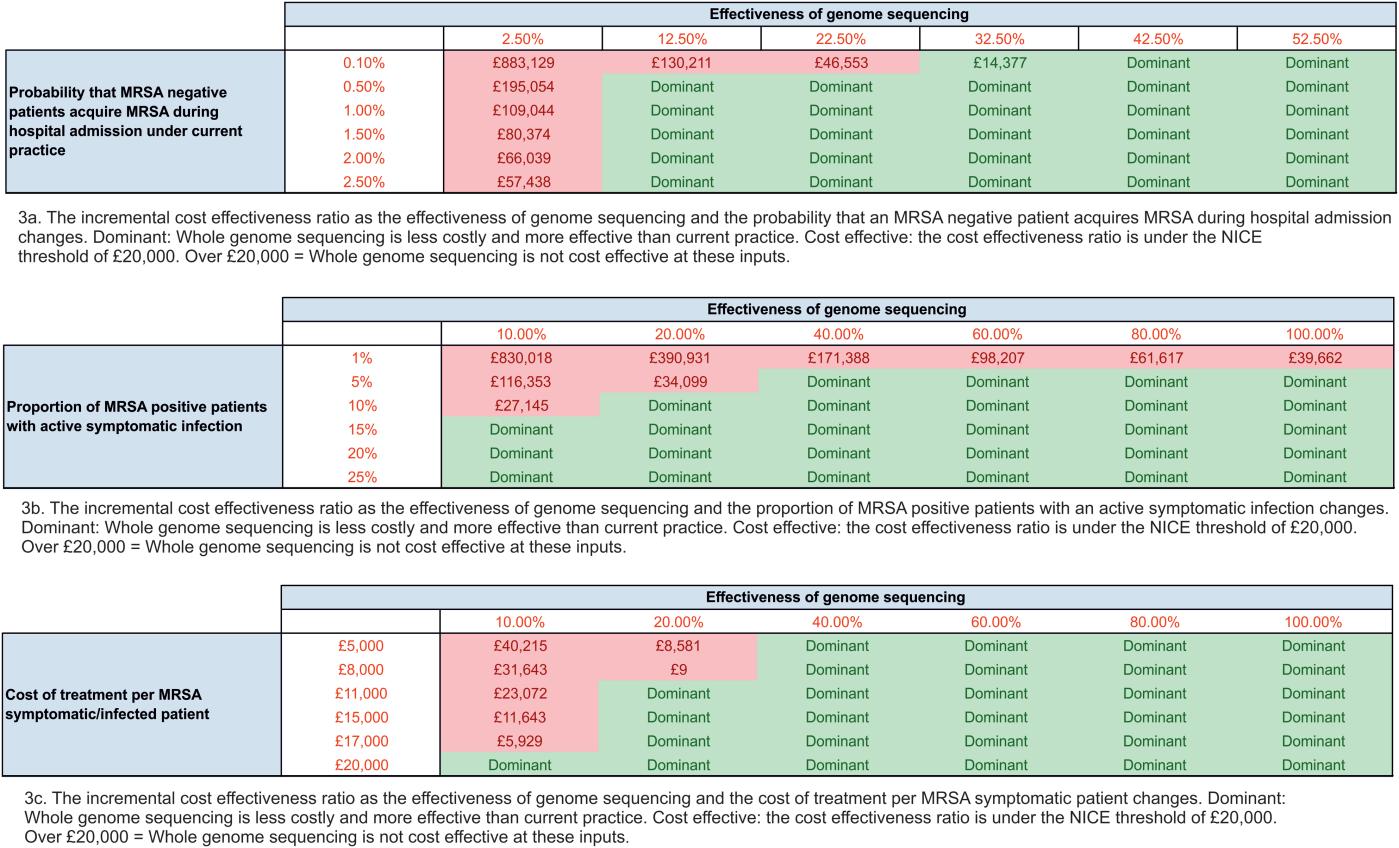
A 2-way sensitivity analysis (cohort of 65 000 patients). Abbreviations: MRSA, methicillin-resistant *Staphylococcus aureus*; NICE, National Institute for Health and Care Excellence.

The 2-way sensitivity analysis presented in Figure 3 provides an overview of how the incremental cost-effectiveness ratio changes when the key drivers of the cost-effectiveness results are varied simultaneously. Whole-genome sequencing was estimated to be cost-effective in the majority of scenarios that have been presented. For example, sequencing was expected to be cost-effective as long as the effectiveness was over 30%, regardless of the probability that a MRSA-negative patient would acquire MRSA during their hospital admission. The effectiveness could drop to 10% and sequencing would still be expected to be cost-effective, as long as the probability that a MRSA-negative patient would acquire MRSA during their hospital admission was over 0.4%.

## DISCUSSION

Our model is the first to estimate the cost-effectiveness of routine whole-genome sequencing of MRSA as an infection control tool. This indicated that routine MRSA sequencing would result in fewer MRSA cases (both carriage and clinical infection, involving any organ or body site), and have a small, positive impact on health-related quality-of-life on a per-patient basis (regardless of whether they have or are tested for MRSA). This technological application is also likely to be cost-saving, assuming the relative reduction in MRSA cases is 90%. Given this assumption, extensive sensitivity analyses supported our cost-saving conclusion, even with large reductions in key parameters.

MRSA screening policies, the prevalence of MRSA, patient characteristics, and infection control practices may vary between different hospitals in England, which limits the generalizability of the results. Despite this, sequencing remained cost-effective when this value was varied in a sensitivity analysis. It must also be noted that the cost-effectiveness of sequencing is dependent upon the prevalence of MRSA colonization in patients upon admission to hospital. A greater prevalence allows for a larger potential for the reduction in MRSA transmission and, hence, a greater benefit associated with sequencing.

Our analyses represent an early, exploratory model that contained several uncertainties in model parameter values. The prospective, observational cohort study was limited to critical care units, and so undersampled the population served by the diagnostic laboratory. This reduced the accuracy of the rates of nosocomial transmission and acquisition of MRSA. MRSA acquisition rates were varied in sensitivity analyses and did not change the cost-effectiveness conclusions of the model. We used a mortality rate of 4.6% for hospital patients who develop a MRSA infection, which represents a composite death rate from all-site infections. This was derived from a recent US study [10], and outcomes may differ in a UK setting. Furthermore, the case mix in this previous study may differ to our patient population; our patients had a very low rate of MRSA bacteraemia [16], which is associated with high mortality [17]. Estimating the baseline QALY of an average hospitalized patient was difficult because of the heterogeneity of the in-patient population as a whole, and we assumed that the health-related quality of life was equal across patients upon entry to a hospital. A sensitivity analysis did not indicate health-related quality of life to be a key driver of the model results. It is also highly likely that we overestimated the cost per case of a MRSA infection, as cost data were based on a study that assumed all patients would be treated within intensive care. These data were used because of the lack of other viable sources from a UK setting [12], but a sensitivity analysis demonstrated that, even at a minimal cost, sequencing would still be cost-effective due to the costs saved through the reduction in colonized MRSA patients.

A previously published comparative effectiveness review of screening for MRSA indicated insufficient evidence to determine the effectiveness of MRSA screening strategies [18]. This did not consider the effectiveness of screening combined with sequencing. A strength of our economic analysis is that it highlights specific data collection requirements of future prospective studies that evaluate the impact and cost-effectiveness of proactive MRSA sequencing.

## Supplementary Data

Supplementary materials are available at *Clinical Infectious Diseases* online. Consisting of data provided by the authors to benefit the reader, the posted materials are not copyedited and are the sole responsibility of the authors, so questions or comments should be addressed to the corresponding author.

ciz480_suppl_Supplementary-Material

## Notes

***Acknowledgments.*** Collection of the data used in the study had ethical approval from the National Research Ethics Service (11/EE/0499), the National Information Governance Board Ethics and Confidentiality Committee (ECC 8-05(h)/2011), and the Cambridge University Hospitals National Health Service Foundation Trust Research and Development Department (A092428).

***Disclaimer.*** The views expressed in this publication are those of the authors and not necessarily those of the Department of Health and Social Care or Wellcome Trust. The researchers were independent from funders and all authors had full access to all of the data (including statistical reports and tables) in the study and can take responsibility for the integrity of the data and the accuracy of the data analysis.

***Financial support.*** This work was supported by grants from the Health Innovation Challenge Fund (grant numbers WT098600 and HICF-T5-342), a parallel funding partnership between the UK Department of Health and Social Care and Wellcome Trust.

***Potential conflicts of interest.*** F. C. has received grants from the Wellcome Trust (grant number 201344/Z/16/Z) and is a paid consultant to Next Gen Diagnostics LLC. A. D., S. M., V. P., and H. D. are employees of York Health Economics Consortium who were commissioned by the University of Cambridge to provide consultancy, develop the early economic model, and write the manuscript. S. J. P. is a consultant to Specific Technologies and Next Gen Diagnostics. All authors have submitted the ICMJE Form for Disclosure of Potential Conflicts of Interest. Conflicts that the editors consider relevant to the content of the manuscript have been disclosed.
