# Supplementary material for: Genomic Surveillance of Methicillin-resistant Staphylococcus aureus: A Mathematical Early Modeling Study of Cost-effectiveness
Source: Clin Infect Dis. 2019 Jun 18;70(8):1613–9. doi: 10.1093/cid/ciz480 (PMC7145999; doi:10.1093/cid/ciz480)
Supplement: ciz480_suppl_Supplementary-Material [file ciz480_suppl_supplementary-material.docx]

**Supplementary Material**

**Threshold Graphs**

Cost per negative screening (cohort of 65,000 patients)


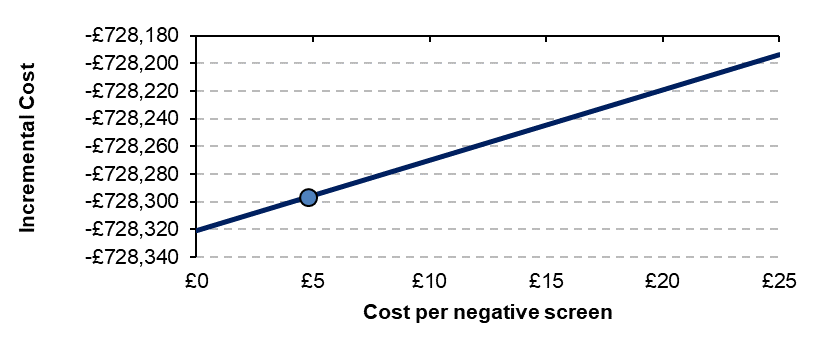


Cost per clinical sample (cohort of 65,000 patients)


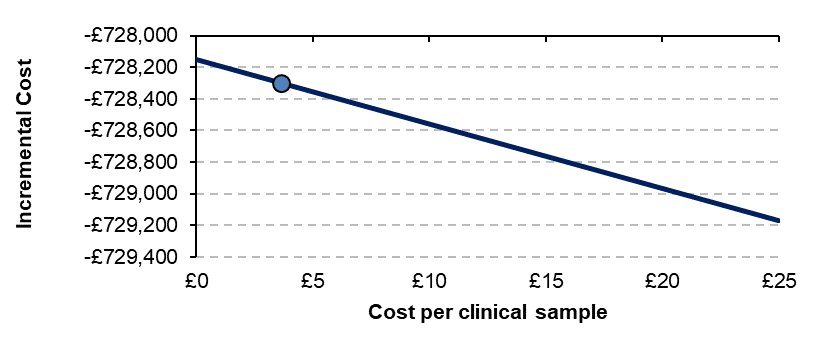


Cost of symptomatic MRSA (cohort of 65,000 patients)


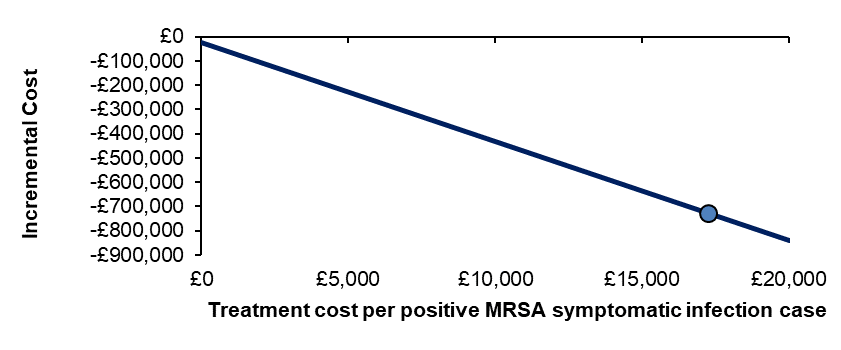


Cost of asymptomatic MRSA (cohort of 65,000 patients)


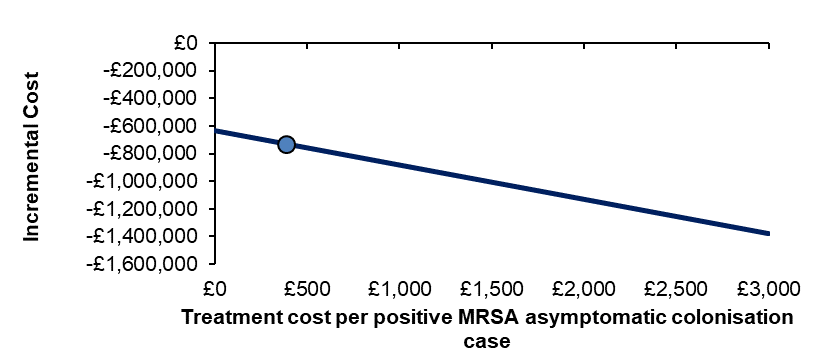


**Scenarios (cohort of 65,000 patients)**

| **Scenario** | **Incremental cost** | **Incremental QALY** | **Cost per QALY** | **Source** |
| --- | --- | --- | --- | --- |
| Base case | -£728,297 | 14 | Dominant |  |
| Mortality at 13% | -£728,297 | 14 | Dominant | [1] |
| 100% screened for MRSA on admission | -£728,321 | 14 | Dominant | [2] |
| Utility decrement per symptomatic MRSA case survived 0.20 | -£728,297 | 8 | Dominant | [3] |
| Utility decrement per symptomatic MRSA case survived 0.24 | -£728,297 | 10 | Dominant | [3] |

**References**

1. Coello R, Jimenez J, Garcia M, et al. Prospective study of infection, colonization and carriage of methicillin-resistant *Staphylococcus aureus* in an outbreak affecting 990 patients. Eur J Clin Microbiol Infect Dis, **1994**; 13: 74-81.

2. Coll F, Harrison EM, Toleman MS, et al. Longitudinal genomic surveillance of MRSA in the UK reveals transmission patterns in hospitals and the community. Sci Trans Med, **2017**; 9: eaak9745.

3. Li J, Ulvin K, Biboh H, Kristiansen IS. Cost-effectiveness of supplementing a broth-enriched culture test with the Xpert meticillin-resistant Staphylococcus aureus (MRSA) assay for screening inpatients at high risk of MRSA. J Hosp Infect, **2012**; 82: 227-233.
